# A novel approach for multi-domain and multi-gene family identification provides insights into evolutionary dynamics of disease resistance genes in core eudicot plants

**DOI:** 10.1186/1471-2164-15-966

**Published:** 2014-11-08

**Authors:** Johannes A Hofberger, Beifei Zhou, Haibao Tang, Jonathan DG Jones, M Eric Schranz

**Affiliations:** Wageningen University & Research Center, Droevendaalsesteeg 1, 6708 PB Wageningen, Gelderland The Netherlands; Chinese Academy of Sciences/Max Planck Partner Institute for Computational Biology, 320 Yueyang Road, Shanghai, 200031 PR China; Heidelberg Institute for Theoretical Studies-HITS, Schloss-Wolfsbrunnenweg 35, 69118 Heidelberg, Baden-Württemberg Germany; Center for Genomics and Biotechnology, Fujian Agriculture and Forestry University, Fuzhou, Fujian PR China; J. Craig Venter Institute, 4120 Torrey Pines Road, La Jolla, CA 92037 USA; The Sainsbury Laboratory, Norwich Research Park, Colney Lane, Norwich, Norfolk, NR4 7UH UK

**Keywords:** Systems biology, Big data, Comparative genomics, Molecular evolution, Plant innate immunity

## Abstract

**Background:**

Recent advances in DNA sequencing techniques resulted in more than forty sequenced plant genomes representing a diverse set of taxa of agricultural, energy, medicinal and ecological importance. However, gene family curation is often only inferred from DNA sequence homology and lacks insights into evolutionary processes contributing to gene family dynamics. In a comparative genomics framework, we integrated multiple lines of evidence provided by gene synteny, sequence homology and protein-based Hidden Markov Modelling to extract homologous super-clusters composed of multi-domain resistance (R)-proteins of the NB-LRR type (for NUCLEOTIDE BINDING/LEUCINE-RICH REPEATS), that are involved in plant innate immunity.

**Results:**

To assess the diversity of R-proteins within and between species, we screened twelve eudicot plant genomes including six major crops and found a total of 2,363 *NB-LRR* genes. Our curated R-proteins set shows a 50% average for tandem duplicates and a 22% fraction of gene copies retained from ancient polyploidy events (ohnologs). We provide evidence for strong positive selection and show significant differences in molecular evolution rates (Ka/Ks-ratio) among tandem- (mean = 1.59), ohnolog (mean = 1.36) and singleton (mean = 1.22) R-gene duplicates. To foster the process of gene-edited plant breeding, we report species-specific presence/absence of all 140 *NB-LRR* genes present in the model plant *Arabidopsis* and describe four distinct clusters of *NB-LRR* “gatekeeper” loci sharing syntenic orthologs across all analyzed genomes.

**Conclusion:**

By curating a near-complete set of multi-domain R-protein clusters in an eudicot-wide scale, our analysis offers significant insight into evolutionary dynamics underlying diversification of the plant innate immune system. Furthermore, our methods provide a blueprint for future efforts to identify and more rapidly clone functional *NB-LRR* genes from any plant species.

**Electronic supplementary material:**

The online version of this article (doi:10.1186/1471-2164-15-966) contains supplementary material, which is available to authorized users.

## Background

Plants have evolved a two-layered innate immune system against microbial and other pathogens [1]. In a first layer of defense, transmembrane pattern recognition receptors (PRRs), usually with extracellular LRR-type domains, recognize pathogen associated molecular patterns (PAMPs) and initiate downstream signaling events including defense gene induction [2], and lead also to cell wall reinforcement by callose deposition and SNARE-mediated secretion of anti-microbial compounds [3, 4]. This is referred to as PAMP- or pattern-triggered immunity (PTI).

Successful pathogens have evolved virulence factors (effectors) that act in the apoplast or inside the host cell to overcome PTI [5]. As a second layer of the innate immune response, many host plant lineages evolved intracellular R-proteins of the NB-LRR type that respond to virulence factors, either directly or through their effects on host targets [6]. Plants producing a specific R-gene product are resistant towards a pathogen that produces the corresponding effector gene product (avirulence factors encoded by *Avr* genes), leading to gene-for-gene resistance [7]. This is referred to as effector-triggered immunity (ETI). Rounds of ETI and effector-triggered susceptibility (ETS) due to novel *Avr* genes on the pathogen side can result in an evolutionary arms-race, generating a “zigzagzig” amplitude of host resistance and susceptibility [1].

R-genes play a major role in defending crops against microbial infection and thus are of great interest in plant breeding programs and efforts to meet increased global food production. In potato, for example, R-proteins of the NB-LRR type confer resistance to the oomycete *Phytophthora infestans*, a hemibiotrophic pathogen that causes late blight [8, 9]. In *Arabidopsis*, R-proteins of the NB-LRR type have been studied extensively in terms of molecular function, structural organization, sequence evolution and chromosomal distribution [10–13]. This superfamily is encoded by scores of diverse genes per genome and subdivides into TIR-domain-containing (for TOLL/INTERLEUKIN LIKE RECEPTOR/RESISTANCE PROTEIN) (TIR-NB-LRR or TNL) and non-TIR-domain-containing (NB-LRR or NL), including coiled-coil domain-containing (CC-NB-LRR or CNL) R-protein subfamilies [14, 15]. For example, the TNL type R-protein RPP1 confers resistance to *Hyaloperonospora arabidopsidis* (downy mildew) in *Arabidopsis*[16]. Similarly, the RPS5 CNL type R-protein interacts in a gene-for-gene relationship with the avrPphB effector from *Pseudomonas syringae* to activate innate immune responses [17]. The TNL type R-protein RRS1, in concert with the TNL protein RPS4, confers resistance to the soil microbe *Ralstonia solanacearum* in *Arabidopsis*[18, 19]. The latter also contains a C-terminal WRKY transcription factor-like domain for DNA binding (Bernoux et al. 2008), increasing the number of domains common to NB-LRR clusters to five. This number is further extended by cases with presence of additional, C-terminal domains mediating extended gene function. For example, the *Arabidopsis NB-LRR* locus *CHILLING-SENSITIVE3* (*CHS3 or DAR4*) encodes a mutated allele of a C-terminal LIM-type domain-containing TNL protein, leading to constitutive activation of defense responses and increased chilling susceptibility [20]. The *NB-LRR ADR1-L1* encodes an N-terminal RPW8-domain whose functional importance has previously been reported [21]. However, many *RPW8*-like genes encode transmembrane proteins without NB-ARC-domain but impact on resistance to powdery mildew in *Arabidopsis*[22–24].

TIR- and non-TIR NB-LRR protein clusters share a conserved central NB-ARC-domain including three subdomains (NB, ARC1, and ARC2). Together, these confer ATPase function [25]. The C-terminal part of NB-LRR proteins harbors a leucine-rich repeat (LRR)-domain for recognition of intracellular effector molecules upon infection, leading to a conformational shift within the NB-ARC-domain [26] upon recognition of the corresponding effector or a change in the surveyed plant protein. In the case of the soybean (*Glycine max*) CNL-class R-protein RPSk-1, defense genes are induced upon *Phytophthora sojae* effector recognition. This includes differential regulation of transcription factor activity as previously proposed [27–29].

A genome-wide comparison of multi-gene families in *A. thaliana* Col-0 revealed a high frequency of gene duplication among the *NB-LRR* gene cluster and impact on genomic distribution [30]. For example, 63% of all reported *NB-LRR* genes are members of tandem arrays in both *A. thaliana* (101/159) and *A. lyrata* (118/185) [11]. Notably, *NB-LRR* loci are subject to positive selection [31]. In this context, [11] re-assessed rates of molecular evolution for both sets of tandem and non-tandem (singleton hereafter) genes and found significant differences in selection rates. In this study, we went a step further by distinguishing the frequency of tandem and ohnolog duplicates to *NB-LRR* cluster expansion and diversity within a wider phylogenomics perspective, thereby covering an evolutionary timeframe of approximately 100 MA that corresponds to the radiation of core eudicots [32, 33]. We compared the average rates of molecular evolution for singleton, tandem and ohnolog duplicate *R*-genes. We further provide evidence for strong positive, but significantly different, selection rates acting on all copy classes of *NB-LRR* duplicates, illustrating the impact of gene and genome duplication to the diversification of plant key traits across approximately 100 MA of genome evolution.

To elucidate the dynamics underlying pathway and trait evolution across multiple lineages, it is of paramount importance to identify and distinguish the complete set of orthologous and paralogous loci present within multiple genome annotations in a phylogenetic framework [34]. Two homologous genes are referred to as orthologs if they descend from one locus present in the common ancestor lineage and diverged due to speciation [35]. By definition, orthologous genes are embedded in chromosomal segments derived from the same ancestral genomic region, thus sharing high inter-species synteny between closely related lineages [36]. In contrast, paralogous loci refer to homologs within one lineage and are due to, for example, tandem, transpositional- or whole genome duplications (WGDs) [37, 38]. Large-scale synteny is not observed for paralogs derived from small-scale events like tandem and transpositional duplication. In contrast, paralogs derived from WGDs are located within intra-species syntenic genomic blocks, and can be referred to as ohnologs or syntelogs [39, 40].

Recent analysis of genome-wide ohnolog distribution have revealed a common history of ancient, successive polyploidy events that are a common feature of genome evolution shared by all flowering plant linages [36]. For example, the *Arabidopsis* lineage underwent at least five polyploidy events that we know of, two preceding and three following angiosperm radiation [41]. The most recent WGD event for the *Arabidopsis* lineage is termed At-α and shared by all Brassicaceae including the extant sister clade Aethionemeae [42, 43]. The older At-β WGD is shared by most species in the order Brassicales, but occurred after the papaya lineage split [44, 45]. The more ancient At-y event is a whole genome triplication (WGT) that is shared by most eudicots including all Rosids, all Asterids (including tomato), Grape (Vitales) and more distant and basal clades such as *Gunnera manicata* (Gunnerales) and *Pachysandra terminalis* (Buxales) [46, 47]. In addition to ancient polyploidy events, more recent, species-specific WGDs/WGTs occurred in various lineages, such as genome triplications in *B. rapa*[48] (Br-α WGT), *T. hasslerania* (Th-α WGT) [44, 49] and the Solanaceae Tomato Genome [50]. Hence, the “syntenic depth” (defined as the level of genome multiplicity expected from the multiplication of successive WGDs/WGTs) of the *Brassica rapa* genome is 36x compared to the putative 1x eudicot ancestor (3x due to At- y, 2x more due to At-β, 2x more due to At-α and finally 3x due to Br- α). Under consideration of two polyploidy rounds at or near the origin of angiosperms as well as 2x at or near the origin of seed plants [41], the syntenic depth of the *B. rapa* genome would be expected to be increased to 144x (“rho-mu-delta-ploidy” genome).

Polyploidy events also influence other kinds of duplication, thereby creating a network of factors with mutual influence. In *Brassica rapa (*that underwent an additional species-specific genome triplication event, see above), arrays of tandem duplicate (TD) genes (TAR genes) fractionated dramatically after the Br- α WGT event when compared either to non-tandem genes in the *B. rapa* or to tandem arrays in closely related species that have not experienced a recent polyploidy event [51]. Errors in DNA replication due to template slippage or unequal crossing-over can result in tandem duplication (TD), producing tandem arrays (TAR) of paralog genes in close genomic proximity [52]. It is known that TAR genes are enriched for genes functioning in biotic and abiotic stress [53]. For disease resistance, there are multiple taxa with an evident impact of TD to trait evolution, including members of Brassicaceae [54], Solanaceae [55] and Fabaceae [56].

Evidence is accumulating for the connection of ancient WGD events to birth and diversification of key biological traits. It was made evident that WGD is often followed by a genome-wide process of biased fractionation that preferentially targets one sub-genome to retain clusters of dose-sensitive genes often organized in functional modules [57–59]. In Brassicaceae, WGD shaped the genetic versatility of the glucosinolate pathway [60], a key trait mediating herbivore resistance and thus highly connected to reproductive fitness of the population. Similarly, starch biosynthesis in grasses, origin and diversification of seed and flowering plants as well as increased species survival rates on the Cretaceous–Tertiary (KT)-boundary are hypothesized to be linked to ancient polyploidy events [33, 61–64].

In this study, we utilized an iterative approach by combining blast, HMM modeling and genomic contextual information provided by synteny to determine the fraction of tandem- and whole genome duplicate copies among all (re)annotated full-length *NB-LRR* genes across twelve species in the context of a phylogenomics perspective, based on uniform standards facilitating comparisons. After utilization of duplicate classes, we assessed and compared rates of molecular evolution to describe a complex interplay of TD and WGD events driving R-protein super-family extension, both of which expanded the evolutionary playground for functional diversification and thus potential novelty and success.

## Results

### Determination of protein domain-specific sub-clusters

**Figure 1:**
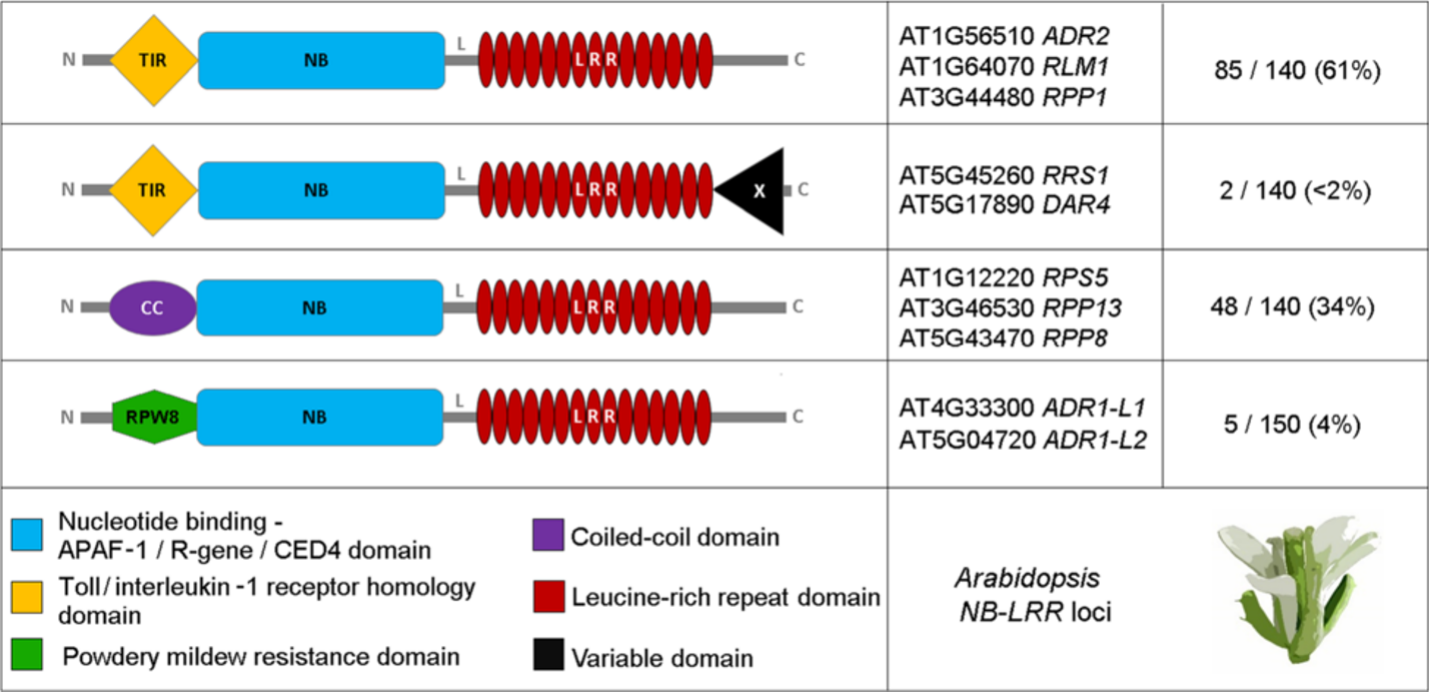
**Domain composition overview for NB-LRR proteins (adapted from** [14]**).** The *NB-LRR* multi-gene family comprises five common and one C-terminal variable domain. Four gene clusters in *Arabidopsis* are defined in this study based on domain compositions. Left: Frequent domain combinations. Middle: Well-characterized class representative in *Arabidopsis thaliana* Col-0. Right: relative abundance of target *NB-LRR* locus class in *Arabidopsis thaliana* Col-0. In case of RRS1, "X" refers to a WRKY TF-like DNA binding domain. In case of DAR4 (CHS3), "X" refers to a LIM-domain.

**Figure 2:**
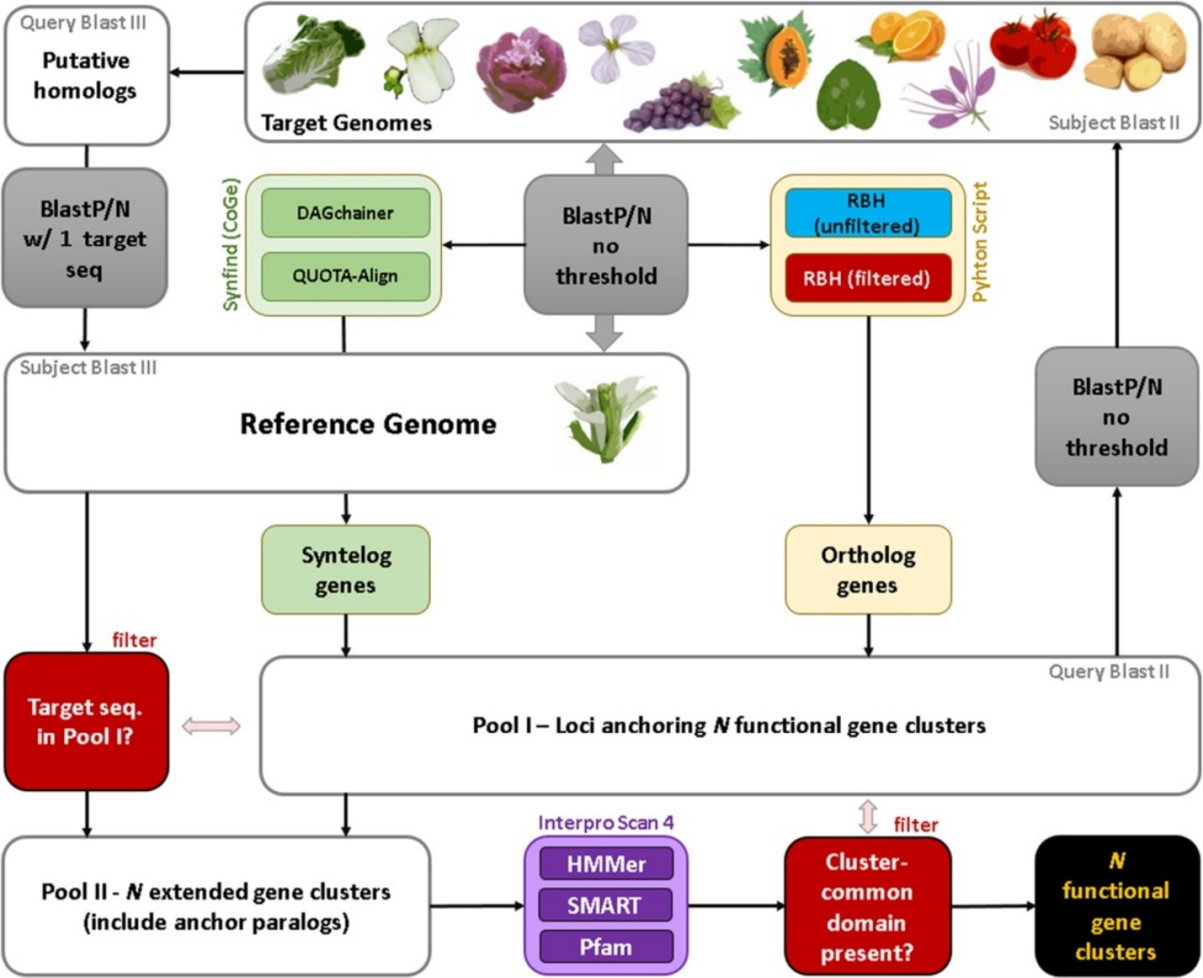
**Custom flowchart showing the integrated approach for the identification of conserved multi-domain protein clusters.** A bi-directional blast screen between a reference genome and n target genomes marks the entry point to the pipeline (grey box in the upper middle). Grey indicates blast screens. Red indicates filtering steps. White boxes indicate pools of FASTA-formatted sequences. Purple indicates Hidden Markov Modelling steps to predict and map protein domains. Green refers to the CoGe system (see Methods). Ochre indicates custom python scripts. Flowchart starts with two-per-genome bidirectional blast screens (middle) and ends with highly accurate functional protein clusters (black, bottom right).

Encoded architecture of *NB-LRR* loci in plants is variable and can comprise up to seven different domains in *Arabidopsis* (Figure 1). In contrast to previous studies [13], we defined functional NB-LRR proteins as composite units sharing both NB-ARC-domain and a LRR-domain signal due to at least one repeat. Hence, TIR-NB-, LRR-only, NB-only or TIR-only proteins are not assigned as NB-LRR proteins by definition. To determine the number of *NB-LRR* loci within a given genome annotation, we combined layers of information provided by sequence homology, protein identity as well as genomic context of target genes in a custom, iterative approach using batch programming (Figure 2).

In the first step, we identified putative orthologous (defined as size-filtered reciprocal best blast hits for both protein and DNA sequences, see Methods) and/or syntenic (based on conserved genomic context, see Methods) “anchor” genes (a) present in the most up-to-date genome annotations of (1) *A. lyrata*, (2) *B. rapa*, (3) *E. parvulum*, (4) *Ae. arabicum*, (5) *T. hasslerania*, (6) *C. papaya*, (7) *C. sinensis*, (8) *V. vinifera*, (9) *N. benthamiana*, (10), *S. lycopersicum* and (11) *S. tuberosum* as well as (b) aligning to any gene present in the (12) *A. thaliana* Col-0 TAIR10 genome annotation. This step resulted in a cluster dataset anchoring every gene family present in *Arabidopsis* to all of the aforementioned lineages, hence providing valuable means for gene identification with any kind of target trait known in core-eudicot plants. Subsequently, we screened for genes encoding (i) a LRR-domain, (ii) a NB-ARC-domain or (iii) a TIR-domain (extended set of target genes defined in this study, see Methods) (Additional file 1). In a second step, we screened for anchor gene paralogs present in every aforementioned genome annotation to form an extended cluster of homologous genes containing at least one of the aforementioned domains (Figure 2). In a third step, we applied multiple machine learning methods (see Methods) to filter false-positives to obtain three highly accurate, functional domain cluster (NB-ARC/LRR/TIR) (Additional file 2). We performed the third (filtering) step three times (once for every aforementioned domain).

**Figure 3:**
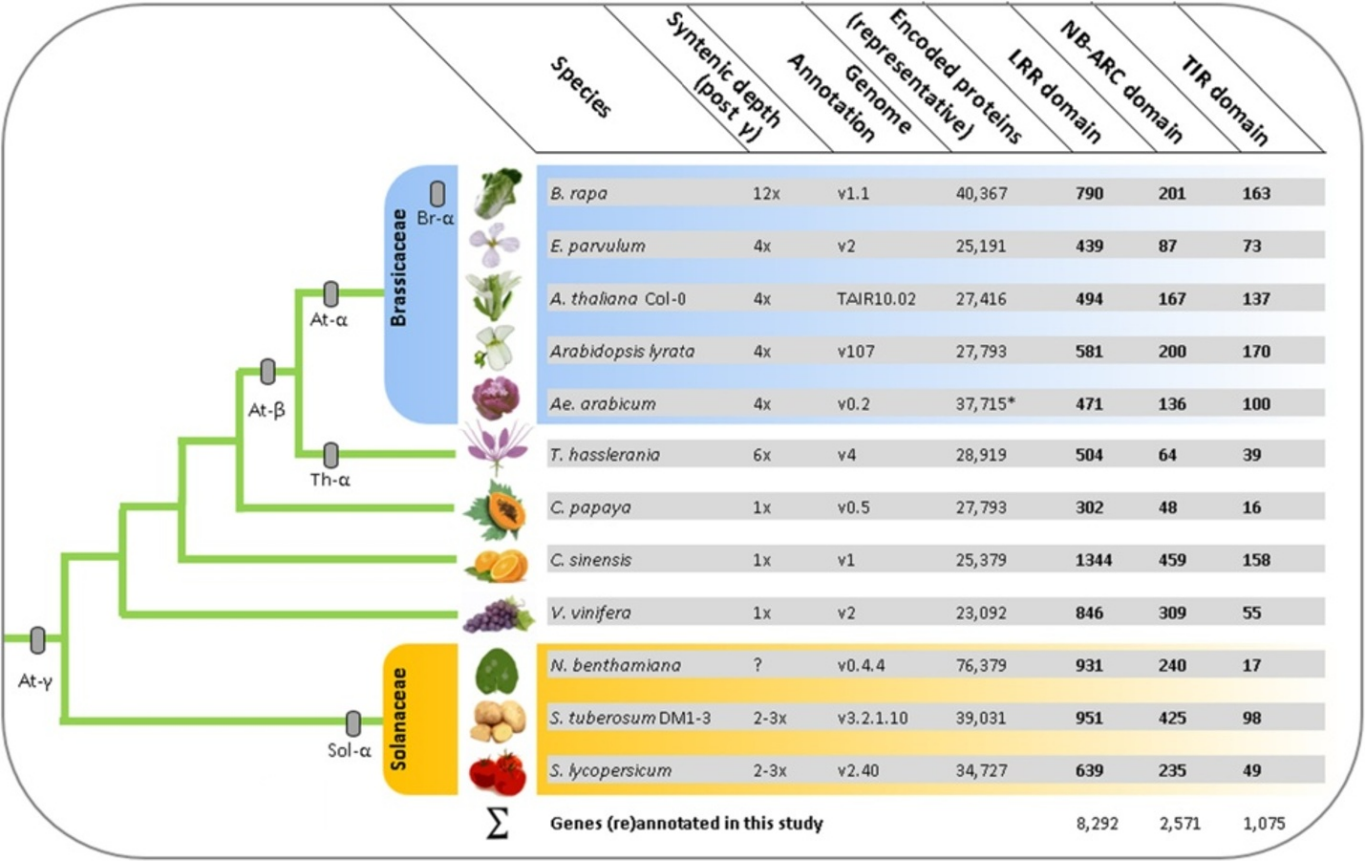
**Results overview of plant Resistance (R)-gene domain (re)annotation.** Shown left are phylogenetic relationships among all analyzed plant lineages and rough placement of whole-genome duplication events. Shown right are numbers of target genes per domain cluster and information on annotation build.

We identified 8,292 genes encoding a LRR-domain in total (Figure 3). Among those, the lowest number of genes containing a LRR-domain is 302 for the *C. papaya* genome annotation v0.5. In contrast, the highest number of genes encoding a LRR-domain is 1,344 for the *C. sinensis* genome annotation v1. Interestingly, both annotations share a syntenic depth of 1x representing the lowest-copy genomes subjected to our analysis (i.e. no major evidence for WGD since At-γ). We identified 2,571 genes encoding a NB-ARC-domain in total (Figure 3). Likewise, the lowest number was found within the *C. papaya* genome annotation v0.5 (48 loci). Again, the highest number of genes encoding a NB-ARC-domain was found in the *C. sinensis* genome annotation v1 (459 loci). We identified a pool of 1,075 genes encoding at least one TIR-domain (Figure 3). Similar to the aforementioned domains, the *C. papaya* genome annotation v0.5 encodes the lowest number of TIR-like loci (16 genes). In contrast to the aforementioned cases, the *A. lyrata* annotation v1.07 (but not *C. sinensis*) contains the highest number of encoded TIR-domains (170 loci). Notably, the syntenic depth of *A. lyrata* is double that of papaya or orange.

### Determination of *NB-LRR*multi-gene family size by overlapping domain-specific sub-clusters

For every analyzed plant species, we determined the multi-gene family size of all annotated *NB-LRR* candidate genes by overlaying each filtered domain clusters. Note that statements about target loci missing or flawed within the gene annotations are beyond the scope of this section, but can likewise be considered *in silico* by applying sequence scaffolds/contigs instead of gene models to our customized pipeline (see Discussion).

**Figure 4:**
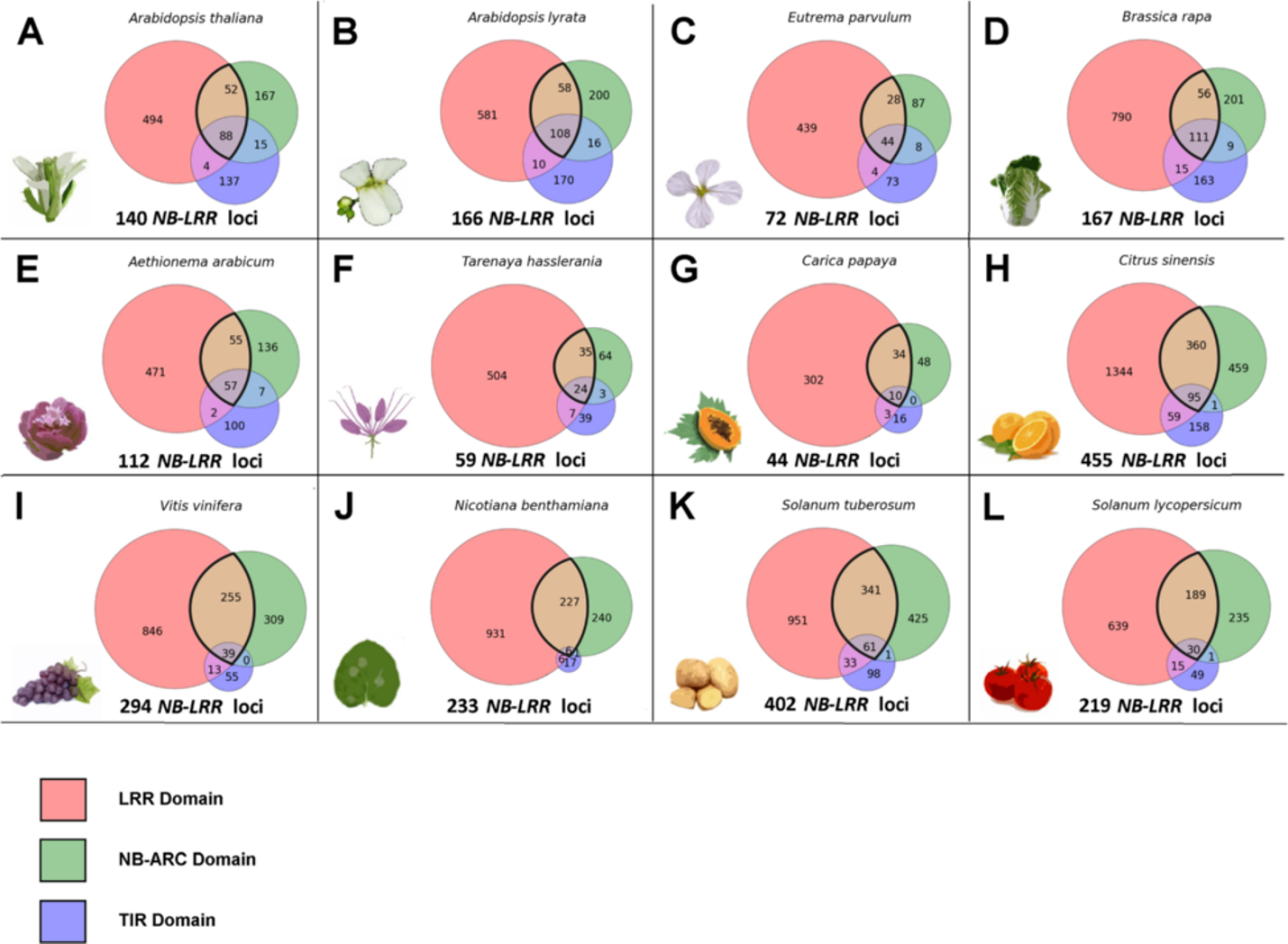
**Area-weighted Venn diagrams illustrating the distribution of three main functional domains common to** ***NB-LRR*** **gene clusters shown for twelve species.** Domain-specific sub-clusters and overlaps are color-coded according to the legend (red: LRR-domain; green: NB-ARC-domain; blue: TIR-domain). Cartoons and italicized Latin names indicate target species: **A**. *Arabidopsis thaliana* **B**. *Arabidopsis lyrata* **C**. *Eutrema parvulum* **D**. *Brassica rapa* **E**. Aethionema arabicum **F**. *Tarenaya hasslerania* **G**. *Carica papaya* **H**. *Citrus sinensis* **I**. *Vitis vinifera* **J**. *Nicotiana benthamiana* **K**. *Solanum tuberosum* **L**. *Solanum lycopersicum*. Please note that we required a *NB-LRR* gene to harbor both NB-ARC- and LRR-domains.

For the *A. thaliana* Col-0 TAIR10 genome annotation, we have found 140 non-redundant *NB-LRR* loci (Figure 4A). Previous studies found 166 [65], 178 [13], 174 [66, 67] and 138 [11]*NB-LRR* loci present in the model plant. In contrast, TAIR10 domain annotation efforts reported 127 target loci [68]. The differences in our study resulted from usage of the updated TAIR10.02 annotation and more stringent criteria; namely the exclusive combination of machine learning with sequence identity and consideration of the genomic context (e.g. synteny). For example, we focus on protein-coding genes only and ignore non-functional (i.e. pseudogenized) loci due to the scope of this study to provide information relevant for breeding of gene-edited crops. Moreover, we defined NB-LRR proteins as sharing both NB-ARC- and LRR-domains, whereas many previous studies score anything as a *NB-LRR* gene that partially aligns to any one domain common to the cluster (i.e. TIR-only, NB-only, LRR-only genes).

For the *A. lyrata* genome annotation v0.2, we identified 166 non-redundant *NB-LRR* loci (Figure 4B). Previous studies reported evidence for 182 [67] and 138 [11]*NB-LRR* loci present in the *A. lyrata* genome assembly. Chen et al. score pseudogenes as well as loci that do not contain both NB- and LRR-domains, leading to the higher number of target genes than reported in this study [67]. The difference between our results and those of Guo et al. is likely due to false-negative target genes with a divergence level that cannot be recognized by their applied HMM-generated NB-ARC consensus sequence [11]. We were able to score these more divergent loci using synteny data anchoring locus determination and subsequent *de novo* domain prediction using a combination of 14 HMM algorithms (see Methods). For example, the *A. lyrata* locus fgenesh1_pg.C_scaffold_8000651 displays only moderate homology (e-value: 1e-34) to the closest related sequence in *A. thaliana,* a P-loop-containing nucleoside triphosphatase that is not defined as *NB-LRR* locus. However, we found both NB-ARC- and LRR-domain within that gene in *A. lyrata*.

For the crop plant *B. rapa* (genome annotation v1.1), we found 167 non-redundant *NB-LRR* candidate genes (Figure 4D), while previous studies reported a sum of 92 [69] and 206 [70] target loci. The latter number includes proteins without LRR-domain (for example TIR-NB or CC-NB). Removing those, Yu and coworkers identified 139 genes encoding both NB-ARC- and LRR-domains, 28 less than we proposed. This differences may be due to our consideration of synteny and application of 14 different HMM algorithms, whereas Yu and coworkers employed HMMER V3.0 only [70]. Note that Mun and coworkers [69] did not have the whole genome assembly available, and hence identified R-proteins based on 1,199 partially redundant BAC clones mostly from a single chromosome. The authors acknowledge a significant degree of sequence redundancy within the available dataset that covers 19-28% of the *B. rapa* genome only. Likewise, [69] performed *ab-initio* gene annotation based on the fgenesh algorithm only [71], and solely use protein sequence homology (based on blastp) for R-protein homolog identification. In contrast, we used the whole gene-space assembly (including every to-date annotated protein-coding gene) as well as three layers of information for homolog identification (see Methods).

To our knowledge, we performed the first analyses of R-proteins for *E. parvulum, Ae. arabicum, T. hasslerania* and *N. benthamiana.* For the extremophile saltwater cress *E. parvulum* (previously known as *Thelungiella parvula*, genome annotation v2), we found 72 non-redundant *NB-LRR* loci (Figure 4C). For *Ae. arabicum*, the extant sister lineage to all other mustard family members (genome annotation v0.2), we identified 112 non-redundant *NB-LRR* loci (Figure 4E). For the *T. hasslerania* genome annotation v4 (previously known as *Cleome spinosa*), we identified 59 non-redundant *NB-LRR* loci for this species (Figure 4F), that underwent a lineage-specific genome triplication event (Figure 3) and has been established as the mustard family outgroup [44, 49, 72]. For the Solanaceae and tobacco relative *N. benthamiana*, we identified 233 non-redundant *NB-LRR* proteins (Figure 4 J). Notably, *N. benthamiana* is widely used as system for transient over-expression and silencing of various genes involved in plant innate immunity to elucidate downstream signaling events after PAMP-mediated priming. In this context, our results provide accurate mapping of all *NB-LRR* –like sequences encoding functions characterized in *A. thaliana* down to the *Nicotiana* gene-space assembly (Additional file 2), thereby facilitating adjusted planning of aforementioned experiments and better understanding of results in the Solanaceae.

For the crop plant *C. papaya* (genome annotation v0.5), we identified 44 non-redundant R-proteins of the NB-LRR type (Figure 4G). Among all species we have analyzed so far, the papaya gene-space assembly encodes the lowest number of R-gene candidates. We again acknowledge the possibility of incomplete gene annotations in this context (see Discussion). However, the low gene count of the *NB-LRR* locus family was previously revealed for the available papaya genes set [73]. The authors found 54 target loci using a combination of tblastx [74] and the pfam HMM algorithm to search for the pfam NB (NB-ARC) family PF00931 domain [75]. The difference in gene-family size estimates is due to an updated genome annotation we have used, as well as more stringent criteria for target gene scoring (i.e. NB-LRR proteins are defined as sharing both NB-ARC- and LRR- domains, see above).

Our analysis revealed 455 non-redundant loci of the *NB-LRR* type for the crop plant *C. sinensis* (orange) (Figure 4H). Evidence for the high R-gene count in orange has been noted previously. For example, the plant resistance gene database (prgdb) lists 3,230 R-genes (including LRR-domain-containing receptor-like kinases/proteins) for this crop plant [76], many of which are redundant. To our knowledge, our study comprises the first efforts to cross-reference both NB-ARC- and LRR-domains among R-genes in orange.

For grape (*V. vinifera*), we found 294 non-redundant R-proteins sharing both NB-ARC- and LRR-domains (Figure 4I). Previous efforts identified 300 target genes [66]. The differences are due to an updated genome assembly as well as more stringent criteria for *NB-LRR* locus definition.

In addition, we subjected the potato crop (*S. tuberosum* Group Phureja DM1-3) genome annotation v3.2.10 to our customized pipeline for identification of homologous gene clusters. We identified 402 encoded non-redundant NB-LRR proteins within the potato genome (Figure 4 K). Previous efforts identified 438 target genes [77] from the annotated proteins set using the MEME and MAST algorithms [78] as well as 755 target genes for the NB-LRR gene repertoire [79] based on reduced representation analysis of DNA enriched (referred to as “Renseq” hereafter [80–82]). Referring to Jupe et al. [79], we acknowledge the inability of our pipeline to identify genes present in the crop but flawed or missing from the annotation or the assembly. The difference between our value and [77] results from more stringent criteria in *NB-LRR* locus identification. For example, at least 34 of the 438 genes from [77] do not contain both NB-ARC- and LRR-domains, whereas at least two do not contain any of the required domains.

For tomato (*Solanum lycopersicum* Heinz 1706), we have found 219 non-redundant R-proteins of the NB-LRR type (Figure 4 L). Previous studies identified 221 target genes sharing both NB-ARC- and LRR-domains in a very conclusive approach [83]. The minor difference in numbers is due to a different build of the annotation based on the genome version 2.4 (fusion of loci/locus fragments) and illustrates the thoroughness of the corresponding authors work. In contrast, application of Renseq to tomato genomic and cDNA recently identified 355 *NB-LRR* genes, thereby highlighting further potential of improvement for *de novo* genome assembly and annotation. Again, we stress that the error rate of our pipeline depends on the quality of the input data (i.e. genes missing in the assembly or annotation can’t be detected).

In total, we identified 2,363 R-proteins of the NB-LRR type. CDS sequences are appended including translation to protein sequences. (Additional files 3 and 4).

### Localization of genes with both NB-ARC- and LRR-domains and determination of tandem duplicate fractions

**Figure 5:**
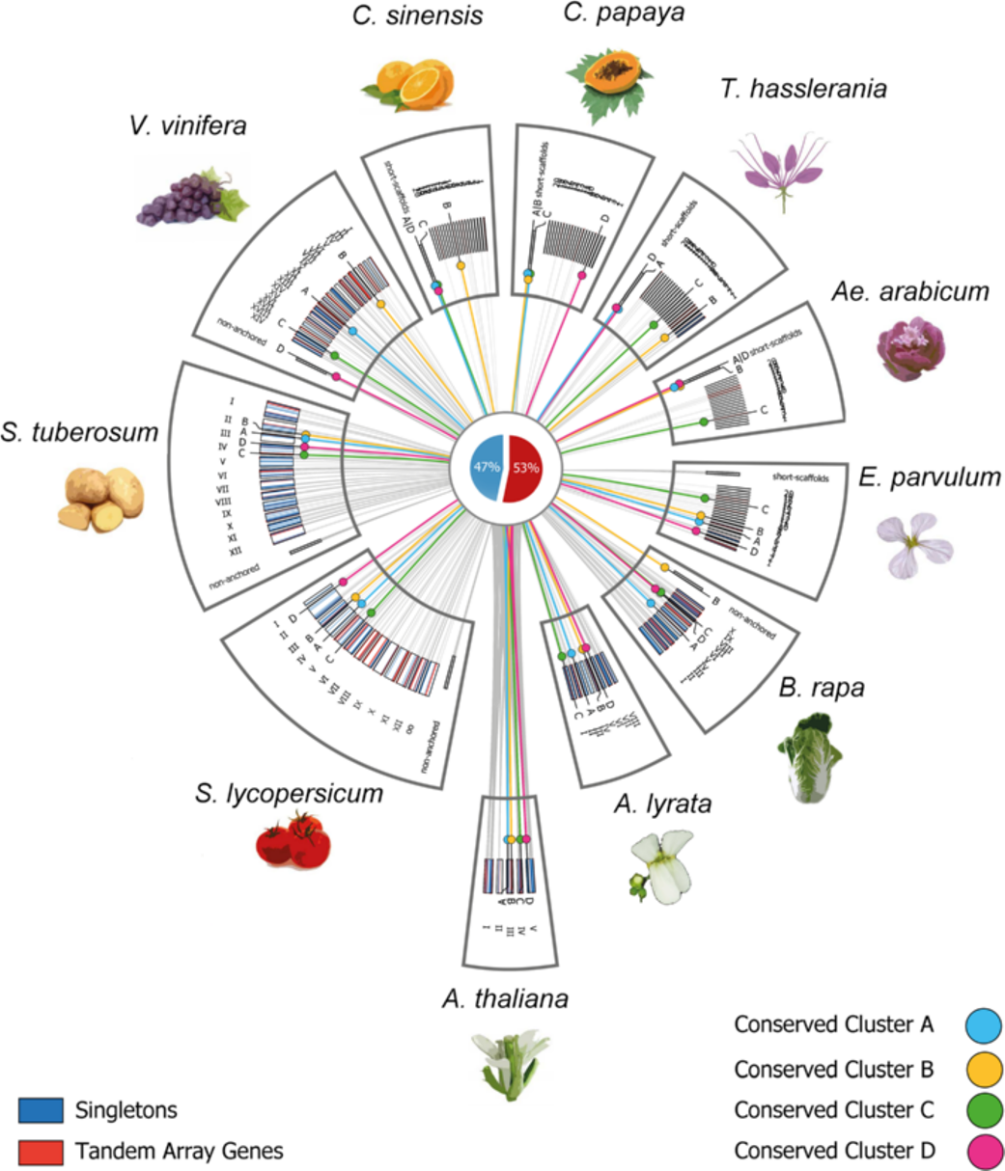
**Circos ideogram with 2,363** ***NB-LRR*** **loci localized on eleven genome annotations.** Latin numbers refer to chromosome pseudo-molecules. Loose scaffolds and contigs not anchored to the genome assembly are shown shifted in radius but not in length scale. For genomes without assembly to the chromosome level, the 20 largest scaffolds are displayed and named in in ascending order with Arabic numbers. Beginning at the bottom block in counter-clockwise orientation, shown are (1) *Arabidopsis thaliana* Col-0, (2) *Arabidopsis lyrat*a, (3) *Brassica rapa*, (4) *Eutrema parvulum*, (5) *Aethionema arabicum*, (6) *Tarenaya hasslerania*, (7) *Carica papaya*, (8) *Citrus sinensis*, (9) *Vitis vinifera*, (10) *Solanum lycopersicum* and (11) *Solanum tuberosum*. Tandem duplicate gene copies are highlighted in red. Singleton genes are highlighted in dark blue. “Conserved Cluster A-D” refers to four distinct *A. thaliana NB-LRR* loci that have been coded in distant colors for easy visual distinction (A: AT3G14470; B: AT3G50950; C: AT4G33300; D: AT5G17860) including ohnologs in all other ten genomes. For genome assembly versions used in this analysis, see Figure 3. Please note that due to the fragmented assembly status of *Nicotiana benthamiana*, all scaffolds of this annotation are below visible length threshold.

We localized all reported *NB-LRR* loci onto the corresponding chromosomes/scaffolds/contigs present in all analyzed genome assemblies except *N. benthamiana* (excluded from Circos plot due to insufficient assembly quality, see Methods). Application of a number of n = 10 allowed gene spacers (see Methods) allowed determination of a global rate of 53% tandem duplicates (Figure 5). Notably, we have found significant differences in tandem array fractions between the analyzed species (up to a factor of 2.8). For example, 70 *NB-LRR* genes present in the *V. vinifera* genome annotation v2 are members of tandem arrays (Table 1). In contrast, the *N. benthamiana* genome annotation v0.4.4 contains only one fourth of tandem duplicates among all present *NB-LRR* loci. The latter represents a fragmented gene-space rather than a genome assembly, leading to a likely under-estimation of tandem duplicates fraction. Hence, the global tandem duplicates fraction drops after inclusion of *N. benthamiana* loci (Table 1). For the mean gene count per *NB-LRR* tandem array, *Aethionema* scores highest. Likewise, the extant mustard family sister clade contains the largest tandem array we found so far. In contrast, the largest orange (*C. sinensis) NB-LRR* tandem array comprises less than half the number of target genes, leading to a very low genome-wide average of *NB-LRR* genes per tandem array for that species (Table 1). Please note that we required presence of both NB-ARC- and LRR-domains for *NB-LRR*-type R-gene curation. Therefore, some of the aforementioned tandem arrays may be further extended due to the presence of partial sequences in close proximity. We do not exclude a biological significance of such fragments *per se*, but set the scope to full-length candidate genes exclusively to obtain a uniform dataset to facilitate comparisons of molecular evolution rates (see below).

**Table 1:** **Array of tandem duplicate copies among** ***NB-LRR*** **loci**

	Number of ***NB-LRR*** genes	Number of tandem duplicates	Fraction of tandem duplicates	Number of tandem arrays	Average number of genes per array	Number of genes in largest array
*B. rapa*	167	92	55%	31	2.9	8
*E. parvulum*	72	37	51%	13	2.8	9
*A. thaliana* Col-0	140	94	67%	32	2.9	8
*A. lyrata*	166	71	43%	23	3.1	9
*Aet. arabicum*	112	71	63%	21	3.4	11
*T. hasslerania*	59	26	44%	10	2.6	6
*C. papaya*	44	32	72%	10	3.2	5
*C. sinensis*	455	136	30%	61	2.2	5
*V. vinifera*	294	206	70%	62	3.3	10
*N. benthamiana*	233	58	25%	26	2.2	5
*S. tuberosum*	402	238	59%	77	3.1	8
*S. lycopersicum*	219	125	57%	40	3.1	7
Σ	2,363	1,186	50%*	406	2.9	7.6

*Difference of value compared to Figure 5 us due to presence on *N. benthamiana.*

Comparison of *NB-LRR* locus-containing tandem arrays* among twelve species.

**Figure 6:**
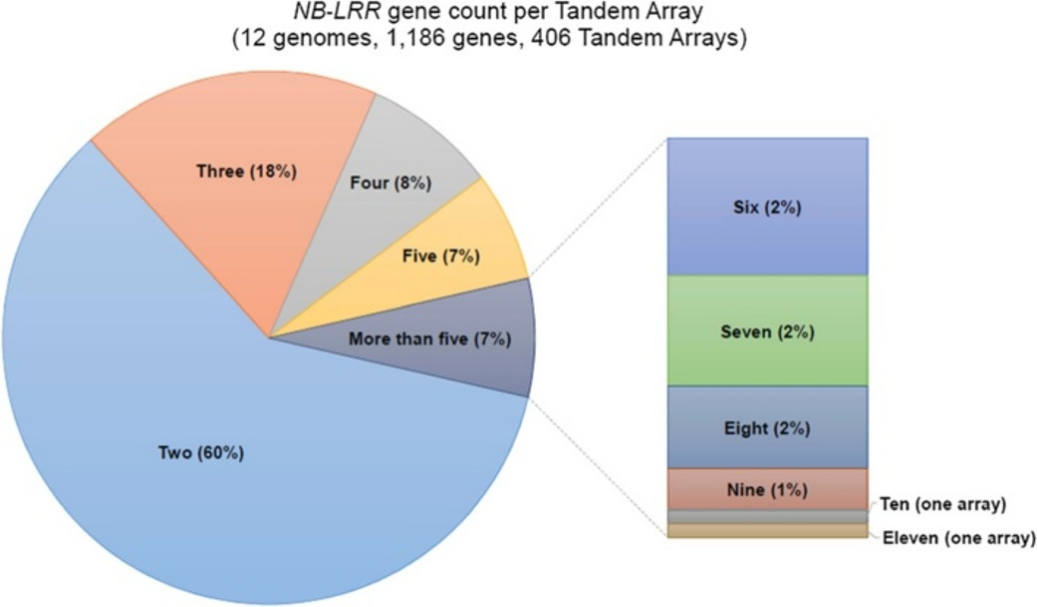
**Gene count listing of full-length** ***NB-LRR*** **candidate gene-containing tandem arrays observed within all twelve analyzed genomes.** 60% of all tandem arrays comprise two duplicate gene copies.

However, our data indicate that both aforementioned outlier situations with high (*Aethionema*) and low (*Citrus*) maximums for gene counts per *NB-LRR* tandem array are outliers beyond the average degree of *NB-LRR* gene tandem array extension. The majority of all 1,191 tandem duplicates (60%) are organized in arrays with two genes only. Three gene members per array occur in less than one fifth of all cases, whereas four, five and more than five genes per array occur with a cumulative frequency below 10% (Figure 6).

### Genome-wide determination of retained ohnolog duplicate fractions and cross-referencing of *NB-LRR*genes

We determined the genome-wide fraction of retained duplicate groups due to ancient polyploidy events (ohnologs), including all *NB-LRR* loci. Screening of pairwise synteny blocks within the analyzed genome assemblies was accomplished using an integer programming approach implemented by the CoGe system for comparative genomics (see Methods) [84]. Due to technical restrictions, this was possible for seven genomes (i.e. minimum requirements in the N50 index, requiring a minimum of approximately 50 kb, see Methods). The high degree of tandem duplicates among R-proteins in all species results in a low degree of retained ohnolog duplicates by definition, because ohnologs mainly comprise groups of two or three duplicates, whereas tandem arrays can have up to eleven members (Figure 6). Notably, the *B. rapa* genome possesses the highest syntenic depth value among all analyzed genome assemblies with 12x in total (Figure 3). Consistently we found the highest fraction of retained ohnolog duplicates both genome-wide and among *NB-LRR* genes present in this crop with in total (Table 2). In contrast, the potato crop (*S. tuberosum*) contains the lowest fractions of retained ohnolog duplicates for both genome-wide average and the set of *NB-LRR* genes (Table 2). On average, about one third of all genes present in the seven analyzed genome assemblies comprise retained ohnolog duplicate groups. This fraction drops among all *NB-LRR* loci. This apparent under-representation of ohnologs among R-proteins highlights the high relative contribution of tandem duplication in R-protein cluster extension for the group of genome assemblies subjected to this analysis (Table 2).

**Table 2:** **Retained ohnolog duplicate copies among** ***NB-LRR*** loci*

	Systenic depth#	Genome-wide average	Among ***NB-LRR*** loci	Ohnolog enrichement
*B. rapa*	12×	53%	42%	No
*E. parvulum*	4×	32%	29%	No
*A. thaliana* Col-0	4×	22%	17%	No
*A. lyrata*	4×	33%	23%	No
*T. hasslerania*	6×	44%	27%	No
*S. tuberosum*	2-3×	10%	5%	No
*S. lycopersicum*	2-3×	19%	16%	No
Σ		30.3%	22.7%	**No**

*Genomes with low assembly quality are excluded from this analysis due to technical reason (see Methods).

#Post-*y* ploidy level.

Species-wise comparison of retained ohnolog duplicates gene pairs among *NB-LRR* loci, shown for seven species*. Genomes with below-threshold mean and median scaffold size (N50 ~ 50 kb) are excluded from this analysis due to technical reasons.

### Uncovering differential patterns of selection acting on subsets of NB-LRR loci pooled according to duplicate origin

**Figure 7:**
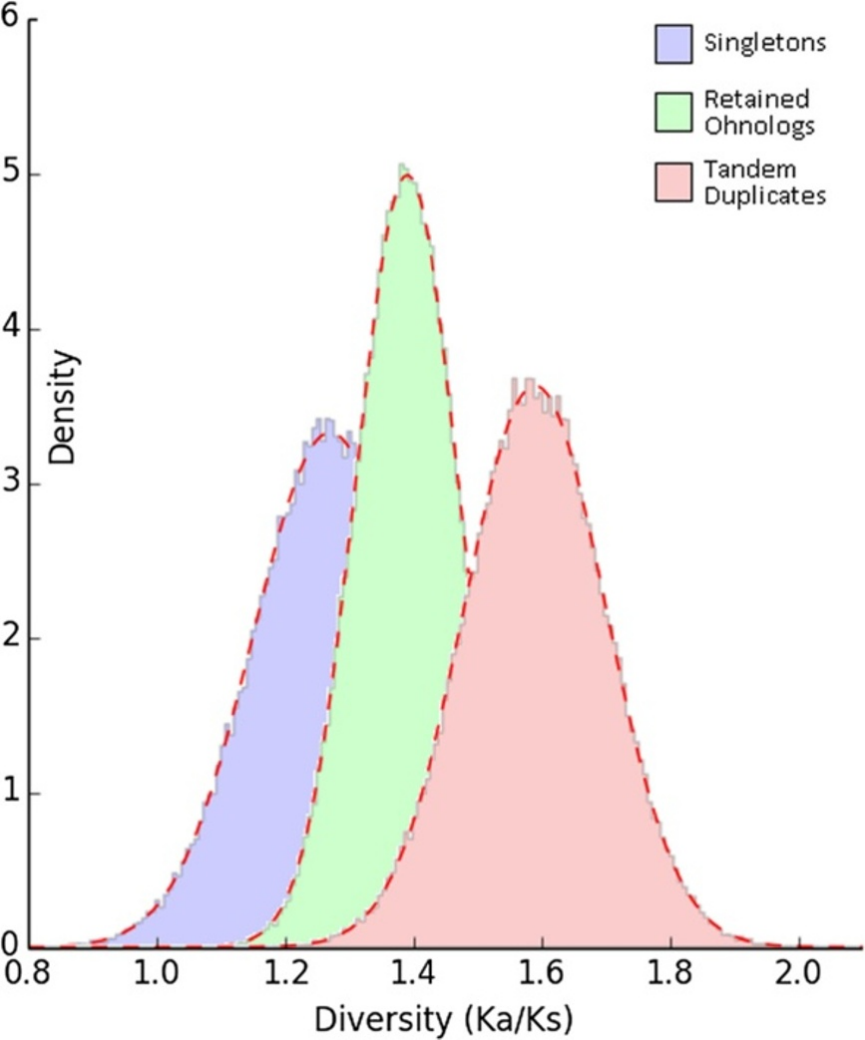
**Selection in action between gene pairs of three major duplicates categories – singletons, tandem duplicates and WGD duplicates (ohnologs).** Strong positive selection following gene and genome duplication of *NB-LRR* loci, as indicated by higher Ka/Ks values.

We performed a genome-wide analysis of molecular evolution acting on all encoded NB-LRR proteins based on both the NB-ARC- and LRR-domain. In a first step, we grouped (a) members of tandem arrays, (b) retained ohnolog duplicates as well as (c) singleton genes (defined as non-tandem array genes without retained ohnolog duplicate). By analyzing non-synonymous substitutions per non-synonymous sites, compared to synonymous substitutions per synonymous site (Ka/Ks ratio or ω, dN/dS), patterns of strong positive selection were uncovered among all three groups. Strikingly, we also found differences in molecular evolution rates among all three groups. Members of tandem arrays evolved fastest with a ω mean of 1.59. In contrast, all analyzed retained ohnolog duplicates evolved with an intermediate rate (ω mean =1.36). We reported the slowest rate of molecular evolution for singleton *NB-LRR* genes with a ω mean of 1.22 (Figure 7). Values for ω above one indicate positive or Darwinian selection, less than one implies purifying (or stabilizing) selection whereas ratios of one are indicative for neutral (i.e. absence of) selection [85].

### Assessing structural dynamics of genomic regions with conserved *NB-LRR*loci

Utilizing the wealth of *NB-LRR* functional and molecular data available in *Arabidopsis* as a reference, we composed a species-wide matrix of R-protein presence/absence based on sequence homology (i.e. filtered/non-filtered reciprocal best blast hits, referred to as “RBH” hereafter) and synteny (Additional files 1 and 2). Among the extended set of 140 distinct *NB-LRR* loci present in the model plant (see above), we found four conserved clusters of “gatekeeper” genes sharing syntenic orthologs across all twelve analyzed genomes (Additional file 1 and Figure 5). Please note that genomic regions displaying conserved synteny across lineages define evolutionary immobile parts of plant genomes [36]. For two among those, functional data are available in *Arabidopsis*, whereas members of the other two gene clusters have not yet been characterized in any of the analyzed plant lineages.

The non-TIR non-CC *NB-LRR* (NL) class R-protein AT3G14460 is a “gatekeeper” because it forms one of four conserved clusters together with all of its aforementioned ohnologs (Additional file 1 and “Conserved Cluster A” in Figure 5). Interestingly, there are yet no functional data available concerning this gene, neither in *Arabidopsis* nor in any of the other eleven analyzed genome/gene-space assemblies.

**Figure 8:**
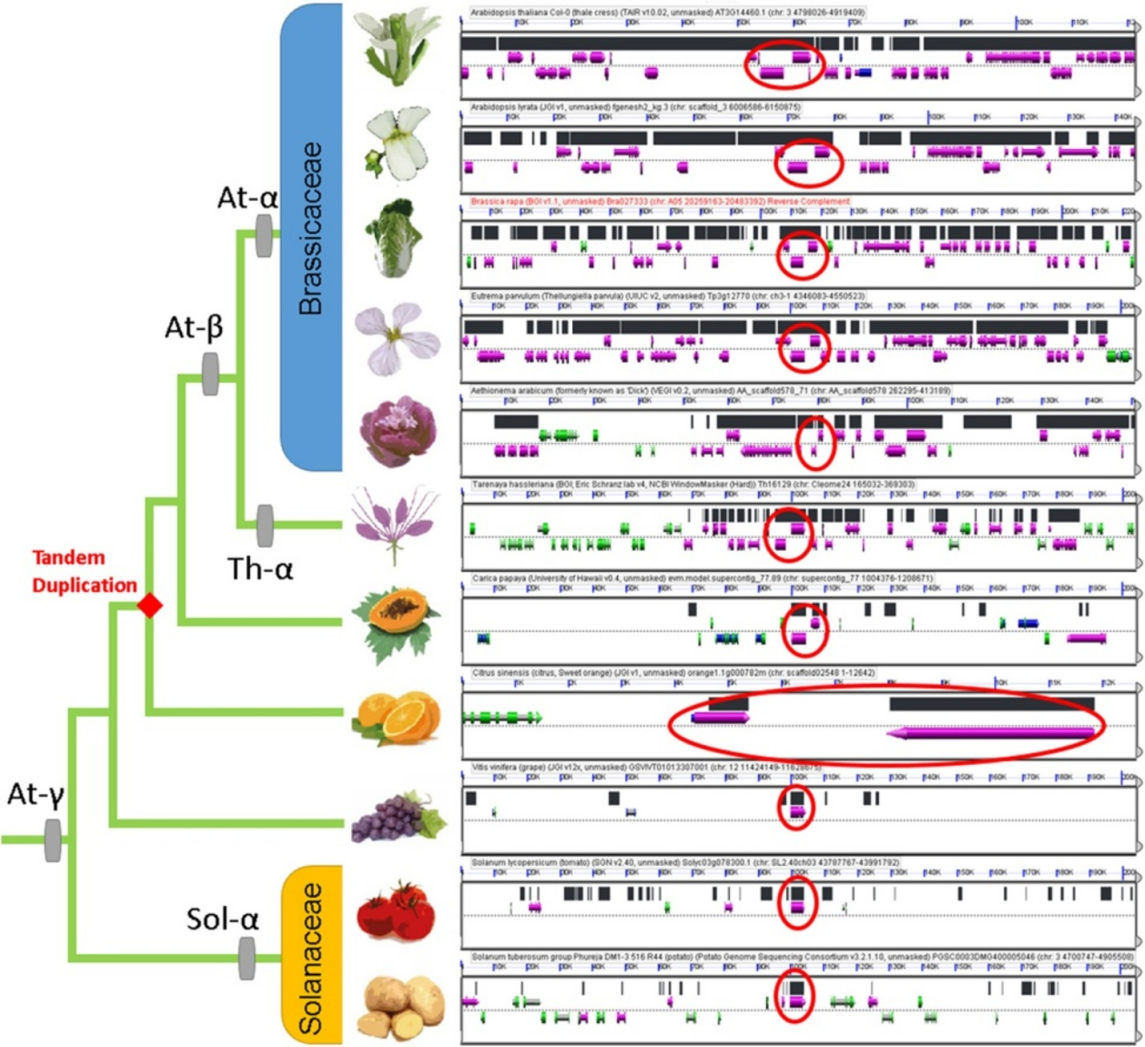
**(B)LastZ eleven-way multiple alignment of conserved cluster A from Figure** 5 **.** Shown left is the phylogenetic relationship among all eleven species (*Nicotiana benthamiana* is excluded from this analysis due to technical reasons). Shown right is the genomic context of the syntenic regions (marked in black). The regions in focus include one *NB-LRR* gene that expanded to a tandem array in the *Arabidopsis* lineage after split of Solanaceae. Diamond indicates a tandem duplication event. Genes not overlapped by HSPs are shown in green. In case of *C. sinensis*, the orthologous genes are harbored by a very small scaffold (~12.6 kb), therefore scaled differently from other panels in GEVO.

For example, this NL-class “gatekeeper” AT3G14460.1 [13, 86] forms syntenic RBH pairs with fgenesh2_kg.3__1571 (*A. lyrata*), Bra027333 (*B. rapa*), Tp3g12770 (*E. parvulum*), AA_scaffold578_71 (*Ae. arabicum*), Th16129 (*T. hasslerania*), supercontig_77.89 (*C. papaya*), GSVIVT01013307001 (*V. vinifera*), Solyc03g078300.1 (*S. lycopersicum*) as well as PGSC0003DMG400005046 (*S. tuberosum*). For *C. sinensis*, the RBH partner orange1.1g000782m is harbored by a very small scaffold (~12.6 kb) with three genes only, making the scoring of gene synteny impossible. However, the locus orange1.1g000782m in turn forms RBH pairs with the aforementioned genes supercontig_77.89 (*C. papaya*) as well as GSVIVT01013307001 (*V. vinifera*), thereby closing the gap in a phylogenetic framework (data not shown). Likewise, the *N. benthamiana* gene NB00009911g0001.1 forms RBH pairs with the aforementioned syntenic orthologs in tomato and grape-vine, overcoming the lack of synteny data for this early-stage draft genome assembly (data not shown). Notably, the underlying locus underwent tandem duplication after grape-vine lineage split, leading to presence of a tandem array in all Brassicales including orange, but an evident singleton gene in Solanaceae and *V. vinifera* (Figure 8).

The *TIR-NB-LRR* (TNL)-class “gatekeeper” locus AT5G17680 is anchoring another group of syntenic orthologs shared by all lineages (Additional file 1, “Conserved Cluster D” in Figure 5). Similarly, this locus lacks evidence on gene function in any of the analyzed plant lineages.

In contrast, conserved clusters B and C are anchored by *ZAR1* (HOPZ-ACTIVATED RESISTANCE 1 or AT3G50950) and the *NB-LRR* gene *ADR1-L1* (ACTIVATED DISEASE RESISTANCE 1 or AT4G33300), that confers pleiotropic effects in *Arabidopsis* innate immunity (Additional file 1, “Conserved Cluster B and C” in Figure 5). *ZAR1* encodes a CC-NB-LRR (CNL) class R-protein of the FLARE group (Flagellin Rapidly Elicited, due to rapid up-regulation following exposure to the PAMP flg22) [87]. ZAR1 confers allele-specific recognition of the *Pseudomonas syringae* HopZ1a type III effector in *Arabidopsis* and acts independent of several gene products required by other R-protein signaling pathways [88]. In contrast, *ADR1-L1* overexpression results in a dwarf phenotype and activation of defense-related gene expression in *Arabidopsis*[21, 87]. Note that *ADR1-L1* encodes an R-protein conferring pleiotropic roles due to function as “helper” NB-LRR that can transduce signals subsequent to specific pattern recognition receptor activation during effector-triggered immunity [89]. Furthermore, *ADR1-L1* encodes the N-terminal RPW8-like domain, whose functional importance in plant innate immunity has been previously reported [23, 90]. Interestingly, the *Arabidopsis* RPW8-like “gatekeeper” was found to be necessary and sufficient to confer induced resistance to powdery mildew in the distant lineage of Solanaceae (*Nicotiana tabacum*) [22]. This case excludes restricted taxonomic functionality and provides additional evidence for functional conservation of syntenic orthologs as defined by “gatekeepers” on a broad phylogenomics range.

In summary, we found four *NB-LRR* genes conserved in sequence as well as linked to structurally immobile parts of the core-eudicot pan-genome. At least one of those confers pleiotropic effects and extended functions in *Arabidopsis* as a “helper-*NB-LRR*” [91, 92]. Although both synteny and sequence conservation across lineages during a timeframe of approximately 250 MA provides strong indications for conservation in function, this may not always be the case. However, we hypothesize that structural stability of the harboring genomic region supports evolution of pleiotropic effects conferred by “gatekeeper“ R-proteins (see below).

## Discussion

The proliferation of high-throughput DNA sequencing and genome informatics approaches enables an accelerated production rate of draft genomes from a wide phylogenetic sampling of plant taxa, highlighting a need for robust methods and a comparative framework for gene and genomic comparisons. We therefore have developed a custom approach to identify functional groups of plant proteins applying a novel and highly complementary combination of available algorithms and datasets. We have applied this to R-proteins and annotated 2,363 loci of the *NB-LRR* type in total. This set contains genes that previously remained un-identified for all species except tomato and potato. For Solanaceae, we stress that re-sequencing approaches based on complexity reduction such as target gene capture have been successfully applied for a similar purpose (referred to as Renseq) [79, 93]. However, it is not unreasonable to assume that the onset of next generation sequencing and genome informatics will continue with acceleration beyond Moore’s law and hence lead to more and better algorithms for *de-novo* generation of gene annotations. Therefore, the added value of the computational pipeline shown in this study will rise with the same rate. For future references, we are working on customization of our approach to make it suitable for application to whole sequence scaffolds/contigs rather than sets of annotated genes/proteins. We intend to generate a computational pipeline for in-silico target gene capture based on scoring of combined hits outside the annotated gene-space within a size-window common to protein-coding genes, thereby overcoming the evident limitations of currently available algorithms for *de-novo* gene annotation (Jupe F, personal communication). The pipeline shown in this study represents the first step towards this goal.

Since tandem duplicates represent the majority of the R-gene duplicates that typically have a higher turnover rate, and additionally most of the R-genes have experienced high birth-and-death rate due to the persistent arms-race with the evolution of pathogen target effectors, most R-genes should have a fairly limited cross-taxonomic coverage [94, 95]. However, a limited set of R-gene clusters are more stable, such as the four gene clusters that we have shown here to be conserved over 100 MA in most (if not all) core eudicot genomes. Could these gene clusters represent shared immunity responses to common pathogens? In addition, the genes in these clusters could also act as “helper *NB-LRR*s”, mediating signal transduction downstream of various different NB-LRR receptors for activation during effector-triggered immunity, thereby leveraging functional constraints as previously made evident for *ADR1* family in *A. thaliana*[91, 92]. Please note that members of the RPW8-domain-containing ADR1-like family have been identified across all angiosperms, providing hints towards relevance of “gatekeepers” in a broad phylogenomics range across the whole angiosperm clade [96] (Zhao and Schranz, unpublished results). More studies need to be done in order to unravel gene function underlying the retention of these unusually “stable” R-gene loci. This is stressed by the fact that (some degree of) functional evidence accumulated for two of our four *NB-LRR* “gatekeeper” functions in *Arabidopsis*; in at least one case ”gatekeeper” R-proteins confer pleiotropic effects as “helper” NB-LRRs. In contrast, such data lacks for the other two “gatekeepers”, notably including one TNL class R-protein. We hypothesize significant potential for extension of gene functional data regarding all four “gatekeeper” loci, either by gene-for-gene resistance towards yet-undiscovered pathogen effectors or by facilitating pleiotropic effects and effector-triggered signaling downstream of other *NB-LRR* genes similar to “helper *NB-LRR*s”. Notably, a combination of both scenarios is evident in *Arabidopsis* and hence not unreasonable to occur in other cases (see above).

We highlight the need for “uniform” standards for comparative studies, such as the method we used in this study that is applicable but by no means limited to R-gene families. In contrast to most past computational pipelines of gene identification that only employ DNA sequence similarity, our approach consolidates multiple tiers of evidence, including the basic protein sequence identity, domain compositions, and genomic context (synteny). Uniform standards also ensure that our gene family member counts are directly comparable with one another, making in-depth studies of the expansion-contraction dynamics of gene families possible. Furthermore, our method allows efficient screening of genome assemblies for near-complete curation of multi-domain and multi-gene family clusters. In the case of *NB-LRR* type R-genes, the resulting raw data provide a detailed overview of nucleotide diversity among all target genes within and between twelve lineages covering the whole core-eudicot clade. Utilizing the wealth of genomics and gene functional data in *A. thaliana*, this leads to species-wise mapping (presence/absence) of every *NB-LRR* sequence present in the model plant. Notably, these data can be used by breeders to identify both target loci as well as small RNA sequence requirements for fast and efficient migration of resistance locus A to organism B using the emerging techniques of genome editing in case restricted taxonomic R-gene functionality doesn’t apply. For example, the particular *NB-LRR* gene conferring the desired resistance can be selected from our curated dataset followed by calculation of the smallest nucleotide distance (or closest related) target gene in the desired organism. The sequence of the small RNA(s) necessary for engineering of nucleases in context of genome editing can be inferred accordingly in order to design a minimum set of experiments necessary and sufficient for gene-editing and thus generating an extended spectrum of resistance in any of the crop subjected to our analysis. However, note that taxonomic restrictions may apply for at least some encoded R-gene functions. Going beyond plant innate immunity, we provide data on a network of anchor genes present in all analyzed genome assemblies, thereby referencing orthologs and paralogs of every gene family present in the model plant *Arabidopsis.* We thereby excel future efforts to extract plant gene function, ultimately necessary for crop improvement and increased rates of global food production.

## Conclusion

We highlight three major findings in this study: (a) higher frequency of tandem gene expansion in R-genes, (b) higher selection ratio in tandem duplicates compared to ohnologs and singletons and (c) evolutionary stable, orthologous R-gene clusters established within structurally immobile parts of plant genomes. Those are likely to indicate a common functional constraint (“gatekeepers”). R-genes typically show an unusually high turnover rate due to strong selection to keep up in a biological arms race with plant pathogens [31, 67]. We suggest such R-genes follow a different evolutionary trajectory than genes with regulatory roles [38]. In this context, the added value of our study lies within the wide phylogenomics scope of the underlying approach. Although similar findings are available in *Arabidopsis*, monitoring dynamics underlying target gene evolution for approximately 100 MA (corresponding to radiation time of the core eudicots) results in higher confidence in the validity of our inferences.

## Methods

### Hardware resources and software prerequisites

All analysis were performed on a commercial Lenovo ultrabook, model Thinkpad X1 Carbon with 8GB RAM and Intel Core i7 3667U CPU (two physical / four virtual cores). The in-house developed perl and python scripts required perl (strawberry v5.18) and python (v2.7) libraries including bioperl (v1.6.910) and biopython (v1.63) modules. The iprscan_urllib.py-script for HMM-based domain annotation (see below) required SOAPy, NumPy and urllib python modules. For blast screens, we employed the stand-alone command line version of NCBI blast 2.2.27+ (ftp://ftp.ncbi.nlm.nih.gov/blast/executables/blast+/LATEST/, last accessed on November 11th, 2014) [74]. For platform-independent coupling and parallelization of all employed scripts and programs, we wrote batch wrappers using the notepad++ editor (http://www.notepad-plus-plus.org, last accessed on November 11th, 2014).

### Genome annotations

The Complete sets of representative genes and proteins for twelve genome annotations were downloaded using http://www.phytozome.net (last accessed on October 15th, 2014) [97]. We included *Arabidopsis thaliana* TAIR10.02 [68], *Arabidopsis lyrata* v107 [98], *Eutrema parvulum* v2 [99], *Brassica rapa* v1.1 [100], *Carica papaya* v0.5 [45], *Citrus sinensis* v1 [101], *Vitis vinifera* v2 [47], *Solanum tuberosum* v3.2.10 [102] and *Solanum lycopersicum* v2.40 (Potato Genome Consortium 2012). *Aethionema arabicum* v0.2 [43]*Tarenaya hasslerania* v4 [49] and *Nicotiana benthamiana* v0.42 [103] genome annotations were made available by the authors.

### Confirmation and extension of the *NB-LRR*multi-gene family in *Arabidopsis thaliana*

We obtained 138 *NB-LRR* genes from [11] and queried them against the TAIR10 *A. thaliana* genome annotation in a blast screen without e-value threshold (forward run). We extracted all target sequences and queried them back against the *A. thaliana* TAIR10 genome annotation with an applied target sequence maximum threshold of two (reverse run). After removal of self-hits, we scored loci as *NB-LRR* genes if they were part of the target sequence pool in the forward run, and aligned to a *NB-LRR* gene as defined by Guo et al. in the reverse run. We thereby created an extended set of *A. thaliana NB-LRR* loci.

### Determination of orthologous gene anchors

In a first step for large-scale *NB-LRR* gene identification, we determined reciprocal best blast hits (RBH) for both (a) protein and (b) coding DNA sequences between *A. thaliana* Col-0 and all other eleven genome annotations in a blast screen without e-value thresholds. Since *NB-LRR* loci can comprise up to seven different domain types connected by partially conserved linkers, the RBH approach can result in false positives due to short but highly conserved alignments of highest-scoring sequence pairs (HSPs) in functionally non-relevant (i.e. structural) parts of the protein. Therefore, we developed a python script to discard RBH pairs with a query/target sequence length ratio below 0.5 and above 2.0. We determined (c) additional, length-filtered RBH pairs for these loci within the aforementioned length ratio scope to form a third line of evidence for orthologous gene detection.

### Syntelog/ohnolog determination

Calculation of pairwise syntenic blocks within and between genomes is based on integer programming [84] but implemented to an easy-to-use web interface termed CoGe platform for comparative genomics (http://www.genomevolution.org, last accessed on November 11th, 2014) [36]. Within all genome assemblies, we determined genes sharing the same genomic context to counterparts in the *A. thaliana* Col-0 genome annotation (defined as ohnologs or syntelogs) using the DAGchainer [104] and Quota-Align [84] algorithms implemented to the “SynMap” function within CoGe. To mask noise generated by successive duplication(s) of ohnolog blocks, we applied Quota-Align ratios for coverage depth consistent with the syntenic depth calculated for each genome annotation. For merging of adjacent syntenic blocks, we applied a threshold of n = 350 gene spacers. For ohnolog gene pairs, we calculated rates of synonymous substitutions (Ks-values) using CodeML of the PAML package [105] implemented to SynMap and applied Ks-value thresholds for ancient WGD events as previously described [39]. For determination of within-species ohnologs (comprising ohnolog blocks due to autopolyploidy events), we proceeded similar with the difference that we queried the target genomes against themselves instead of against *Arabidopsis*, using the “SynMap” function within the CoGe platform for comparative genomics (parameters: gene order = relative/minimum cluster size = 5 genes/maximum chaining distance =20 genes/scoring function = collinear). The latter parameter enforces, together with the maximum chaining distance, scoring dense arrangement of collinear gene pairs as previously described [36, 106] and provides a *de facto* density cutoff. Note that gene density cutoffs per Kb/Mb would not be consistent between different synteny runs since values vary greatly across genomes, or even across different regions within the same genome as previously described [36, 106]. For the lineage-specific WGD events known for *B. rapa*, *T. hasslerania*, *S. tuberosum* and *S. lycopersicum*, we set maximum thresholds for Ks value averages of ohnolog blocks (1.5) to eliminate noise of recent duplication events. Due to minimum requirements on assembly quality that apply for usage of SynMap, it was not possible to determine the fraction of ohnolog duplicates for the current gene-space assemblies of *Aethionema*, *Carica*, *Citrus*, *Vitis* and *Nicotiana* with the available algorithms. Synteny of genes within and between lineages was visualized using the GEVO function implemented to the CoGe platform for comparative genomics (see above).

### Determination of anchor paralogs and generation of extended multi-gene family cluster pool

We defined the orthologous gene sets as sum of three groups of RBH pairs (first group: based on length-filtered protein pairs; second group: based on non-length-filtered protein pairs; third group: based on non-length-filtered CDS pairs; see above for length filter criteria). We merged the orthologous gene sets with the ohnolog genes set to create a set of putative homologous loci anchoring all *A. thaliana* gene families in all other analyzed genome annotations (“anchor pool”). In a next step, we performed a blast search without e-value threshold to query all homologous anchor genes against all twelve genomes to determine putative paralogs of the anchor genes set (forward run). We extracted all target sequences and queried them against the *A. thaliana* Col-0 TAIR10 genome annotation with a target sequence maximum threshold of two (reverse run). After removal of self-hits, we scored loci as *NB-LRR* if they aligned to any member of the extended NB-LRR locus cluster in *A. thaliana* (see above). We defined all members of this pool as anchor paralogs if they are not present within the set of homologous anchor genes (see above), thereby creating a highly accurate super-cluster of *NB-LRR* genes across twelve genomes.

### Hidden Markov modeling and prediction of protein domains

The above-mentioned extended multi-gene family cluster of *NB-LRR* genes is based on both sequence homology and genomic location of its members. However, we observed an erosion of synteny across lineages relative to their phylogenetic distance. Furthermore, DNA sequence homology decreases with phylogenetic distance due to wobble rules for the third codon position. Likewise, the protein sequence homology between distant multi-gene family members can decrease due to synonymous substitutions of amino acids belonging to the same chemical class (i.e. aliphatic, aromatic or indolic). Therefore, we applied a final filtering step to remove false-positives from the extended *NB-LRR* gene cluster pool across all genomes. Using the iprscan_urllib.py script provided by the European Molecular Biology Laboratory (EMBL, Heidelberg, Germany) (https://www.ebi.ac.uk/Tools/webservices/services/archive/pfa/iprscan_rest, last accessed on November 11th, 2014), we queried every member of the extended *NB-LRR* cluster pool to 14 algorithms that apply Hidden Markov Models for (protein domain) signature recognition (BlastProDom, FPrintScan, HMMPIR, HMMPfam, HMMSmart, HMMTigr, ProfileScan, HAMAP, PatternScan, SuperFamily, SignalPHMM, TMHMM, HMMPanther and Gene3D) [107]. We overcame the one-sequence-at-a-time limitation of the EMBL server by writing batch wrappers for 25x-fold parallelization. To form a second layer of control we additionally tested all target genes for an encoded LRR-domain using the “LRRfinder” -algorithm version 2.0 available at http://www.lrrfinder.com/ (last accessed on November 11th, 2014) [108]. As a result, we mapped all protein domains present in the putative multi-gene family cluster onto their genes in less than a day, and discarded all false positive genes (i.e. genes not coding for at least one cluster-common domain). Final referencing of proteins with both NB-ARC- and LRR-domains was performed using a multi-vlookup array function in MS excel 2013.

### Determination of tandem duplicate gene copies

To determine the fraction of tandem duplicate gene copies, we queried the complete protein annotation of every genome assembly against itself in a blast screen without any e-value threshold and filtered our final set of target sequences from above outside a window of n = 10 allowed gene spacers in both directions from the query sequence as previously described [53]. Likewise, we have filtered hits with genomic location on distant chromosomes/scaffolds/contigs to avoid false-positive scoring of transpositional duplicates.

### Multiple protein alignments

To generate multiple alignments of protein sequences, the stand-alone 64-bit version of MAFFT v7 was employed (http://mafft.cbrc.jp/alignment/software/, last accessed on November 11th, 2014) [109]. First, all NB-LRR proteins were aligned species-wise together with the HMM-generated consensus sequence of the NB-ARC-domain (available at http://niblrrs.ucdavis.edu/At_RGenes/, last accessed on November 11th, 2014) as well as the LRR-domain (available at http://smart.embl.de/smart/do_annotation.pl?DOMAIN=SM00370, last accessed on October 15th, 2014) using the command line mafft.bat --anysymbol --thread 4 --threadit 0 --reorder --auto input > output. Mesquite v2.75 (http://mesquiteproject.org, last accessed on November 11th, 2014) was used with multi-core preferences to trim MAFFT multiple alignments down to the NB-ARC- and LRR-domain blocks. Trimmed blocks were re-aligned using MAFFT with the command line mafft.bat --anysymbol --thread 4 --threadit 0 --reorder --maxiterate 1000 --retree 1 –localpair input > output.

### Codon alignments and determination of substitution rates

Re-aligned NB-ARC- and LRR-domain blocks were transferred to codon alignments using the CDS sequence counterparts and the pal2nal.pl script v14 [110] (http://www.bork.embl.de/pal2nal/distribution/pal2nal.v14.tar.gz, last accessed on November 11th, 2014). Gaps were allowed but manually edited wherever necessary. We allowed unusual symbols and manually edited mismatches between CDS and protein sequences wherever necessary. Synonymous and non-synonymous substitution rates were determined using the “KaKs_Calculator“ software (https://code.google.com/p/kaks-calculator/wiki/KaKs_Calculator, last accessed on November 11th, 2014) [111] including ten substitution rate estimation methods (model averaging was applied). Divergence rates are generally determined between pairwise alignments of homologous sequences. For determination of average divergence rates among singletons (i.e. non-TD non-ohnolog loci), we aligned singleton *NB-LRR* loci with the best non-self blast hit among all target genes within one species. For determination of average divergence rates among retained ohnolog duplicates, we aligned all ohnolog *NB-LRR* loci with the best non-self blast hit among all ohnologs within one species. In case of ohnolog triplets, we only considered the highest-scoring sequence pair (HSP). For determination of average divergence rates among arrays of tandem duplicate *NB-LRR* genes, we aligned the first with the last member of every array, thereby covering the majority of all tandem arrays (see Results). In a control step, we determined average divergence rates for all pairwise combinations within the largest tandem array in every species and did not find significant deviations (data not shown).

### Generation and graphical editing of figures

Ideograms of plant chromosomes/scaffolds/contigs were generated using the circos package (http://circos.ca/, last accessed on November 11th, 2014) [112]. Histograms and Venn-diagrams were generated using the matplotlib package (http://matplotlib.org/, last accessed on November 11th, 2014). Other figures were generated with MS office and graphically edited using the GIMP package (http://www.gimp.org/, last accessed on November 11th, 2014).

### Availability of supporting data

The data sets supporting the results of this article are included within the article and its additional files.

## Electronic supplementary material

Additional file 1: **Syntelogs and orthologs of all** ***Arabidopsis*** **NB-LRR genes across all analyzed species.** (XLSX 12 MB)

Additional file 2: ***NB-LRR*** **gene IDs, duplicate classes and closest homolog in** ***Arabidopsis.*** (XLS 394 KB)

Additional file 3: **CDS sequences of identified genes encoding both NB-ARC- and LRR-domains.** (TXT 6 MB)

Additional file 4: **Translated protein sequences of identified genes encoding both NB-ARC- and LRR-domains.** (TXT 2 MB)

## Abbreviations

*A. lyrata*: *Arabidopsis lyrata*
*A. thaliana*: *Arabidopsis thaliana*
*Ae. arabicum*: *Aethionema arabicum*
ARC: APAF-1, R proteins, and CED-4
ATPase: Adenosine triphosphatase
Avr: Avirulence
B. rapa: Brassica rapa
blast: Basic local alignment search tool
*C. papaya*: *Carica papaya*
*C. sinensis*: *Citrus sinensis*
CC: Coiled-coil
CNL: Coiled-coil nucleotide binding site leucine-rich repeats
dS / Ka: Non-synonymous substitutions per non-synonymous site
dN / Ks: Synonymous substitutions per synonymous site
DNA: Deoxyribonucleic acid
*E. parvulum*: *Eutrema parvulum*
EMBL: European Molecular Biology Laboratory
ETI: Effector-triggered immunity
ETS: Effector-triggered susceptibility
eudicot: Eucotyledon
FLARE: Flagellin rapidly induced
HMM: Hidden markov model
HSP: Highest-scoring sequence pair
Kb: Kilobases
KT: Cretaceous–Tertiary
LRR: Leucine-rich repeats
MA: Million years
Mb: Megabases
*N. benthamiana*: *Nicotiana benthamiana*
NB: Nucleotide binding site
PAMP: Pathogen-associated molecular pattern
pfam: Protein families
PRR: Pattern-recognition receptor
PTI: PAMP-triggered immunity
RBH: Reciprocal best blast hit
RNA: Ribonucleic acid
R-proteins: Resistance proteins
RPW8: Resistance to Powdery Mildew 8
*S. lycopersicum*: *Solanum lycopersicum*
*S. tuberosum*: *Solanum tuberosum*
SNARE: Soluble N-ethylmaleimide-sensitive factor attachment protein receptor
*T. hasslerania*: *Tarenaya hasslerania*
TAIR10: The *Arabidopsis* information resource version 10
TAR: Tandem array
TD: Tandem duplication
TIR: Toll/interleukin like receptor
TNL: TIR-domain-containing nucleotide binding site/leucine-rich repeats
*V. vinifera*: *Vitis vinifera*
WGD: Whole genome duplication
WGT: Whole genome triplication.
